# Self-perceived burden predicts lower quality of life in advanced cancer patients: the mediating role of existential distress and anxiety

**DOI:** 10.1186/s12877-022-03494-6

**Published:** 2022-10-17

**Authors:** Lin Xiaodan, Xu Guiru, Chen Guojuan, Xiao Huimin

**Affiliations:** School of Nursing, Fujian Medical University, Fuzhou, China

**Keywords:** Self-perceived burden, Existential distress, Anxiety, Quality of life, Mediating analysis

## Abstract

**Background:**

Self-perceived burden (SPB) is an important predictor of quality of life (QoL) in patients with advanced cancer. However, the mechanism how SPB affects patients’ QoL remains unclear. This study aimed to examine the potential mediating roles of existential distress (ED) and anxiety in the relationship between SPB and QoL.

**Methods:**

A multicenter cross-sectional study was conducted. 352 advanced cancer patients were recruited from three hospitals in southeast of China. The Self-perceived Burden Scale, the Existential Distress Scale, the Hospital Anxiety and Depression Scale, and the Quality-of-Life Concerns in the End of Life Questionnaire were adopted to collect data. Hayes’s bootstrapping method was used to analyze the data.

**Results:**

SPB was negatively associated with QoL (*P* < 0.01). ED and anxiety partially mediated the relationship between SPB and QoL (*P* < 0.01). Moreover, ED had direct effects on anxiety, and sequentially QoL (*P* < 0.01). The serial multiple mediation model of SPB accounted for 73.25% of the variance in QoL in advanced cancer patients.

**Conclusions:**

ED and anxiety are important mediating factors between SPB and QoL in advanced cancer patients. To improve patients’ QoL, comprehensive interventions for reducing anxiety and ED are highly recommended in clinical practices.

**Supplementary Information:**

The online version contains supplementary material available at 10.1186/s12877-022-03494-6.

## Introduction

It is reported that the global cancer burden is expected to be 28.4 million cases in 2040, a 47% rise from 2020 [1]. Among them, there is an 11-fold increased incidence of cancer in the population aged 65 years and over compared to younger adults [2]. An estimated 9 million people die from cancer annually, most of whom are in the advanced stages of the disease. Advanced cancer patients, whose cancer is at stage III or IV, are particularly exposed to deteriorating physical conditions, significant psychological trauma, and impending death [3, 4]. Thus, they often have to depend on families in terms of instrumental, emotional, and economic support, leading to a sense of self-perceived burden (SPB) and poor quality of life (QoL) [5]. However, how SPB affects QoL of advanced cancer patients remain unclear.

"QoL is a global measure of well-being that encompasses the impact of challenges from the patient's point of view" [6]. Chen's concept model of existential distress (ED) [7] and Walster’s equity of theory [8] further reveal the relationship between SPB, ED, anxiety, and QoL. The concept model of ED indicates that SPB is one of the antecedents of ED, which can result in a series of negative consequences among advanced cancer patients, such as low QoL and anxiety. According to the equity theory [8], advanced cancer patients feel SPB for they losing the ability to keep balance in receiving and giving help, resulting in negative emotions, such as anxiety. Theoretically, ED and anxiety could be the mediators of the relationship of SPB and QoL.

Numerous studies have identified the predictors of QoL among advanced cancer patients. Among them, SPB seems to be a fundamental factor related to QoL [9]. A previous study indicated that cancer patients with low SPB reported a higher QoL [10]. It was further confirmed by another study, which showed that advanced cancer patients having higher level of SPB were more likely to report poorer QoL [11]. It may be because that patients with SPB tend to suppress requests and emotions to mitigate their feelings of SPB and minimize the burden of the care to their families [12]. ED was another significant factor of QoL, as it could trap individuals in a subjective incompetence state [13]. Kissane et al. found the negative relationship between ED and QoL in advanced cancer patients [14]. Consistently, Ghiggia et al. found that ED emerged as a risk factor for QoL in advanced cancer patients [15]. Anxiety is also revealed as a significant factor for QoL. As a response to a threat like cancer, anxiety could impair QoL, including physical, emotional, and social dysfunction [16]. A previous study reported that higher anxiety was collated with lower QoL in advanced cancer patients [15]. Another study also concluded that cancer patients with higher anxiety at the start of treatment is associated with decreased QoL at the beginning of therapy and post-diagnosis [17].

The association among SPB, ED and anxiety is described in previous studies. Evidence suggested that SPB was a primary determinant of ED [18]. It was supported by Barbosa et al., who found ED was related to the perception of being a burden on others [19]. Recently, Chen’s study also reported that SPB could cause ED in advanced cancer patients [7]. SPB also was found to be a risk factor for anxiety. Advanced cancer patients, who felt they imposed hardship on others without being unable to restore, would suffer from extensive psychological consequences, such as anxiety [20]. It was demonstrated that the feeling of a burden on others in advanced cancer patients was negatively related to anxiety [21]. It was also confirmed that worrying being a source of burden strongly correlated with psychological well-being in cancer patients [5]. In addition, the relationship between ED and anxiety is also demonstrated previously. For instance, a study conducted in Spain found that anxiety was positively associated with ED [22]. It was supported by another study performed in Germany, which showed that advanced cancer patients who have inadequately treated anxiety were at increased risk for ED [23].

**Fig. 1:**
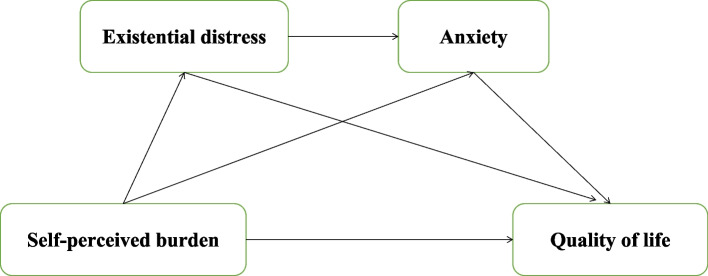
Conceptual serial mediation model

Although many studies have found the significant relationship among SPB, ED, anxiety and QoL, to date, no study has examined the effects of ED and anxiety on the relationship between SPB and QoL. Based on the above mentioned theoretical and empirical background, this study proposes a conceptual serial mediation model (Fig. 1). It is hypothesized that SPB exerted a direct on QoL, and an indirect mediated by ED and anxiety. Moreover, we hypothesized that ED and anxiety would both act as mediators individually, as well as in a sequential manner. This study aims to provide a new insight into the mediating role of ED and anxiety between SPB and QoL among advanced cancer patients.

## Methods

### Design, participants, and settings

This study employed a multicenter cross-sectional design. Overall, 352 participants were recruited for this study from June 2020 to January 2021 from three hospitals in Fujian, southeast of China. The inclusion criteria were as follows: (a) diagnosed with advanced cancer at stage III or IV according to TNM Staging System; (b) aged 18 years old or above; (c) aware of diagnosis and treatment; (d) without verbal communication impairment or cognitive impairment; and (e) capable of giving written informed consent. The exclusion criteria were as follows: (a) severely disabled or critically ill (Karnofsky Performance Status, KPS < 40%); and (b) experiencing a visual, hearing, or psychiatric disorder.

The following formula for descriptive cross-sectional study was used to determine the sample size: *n* = t^2^ p(1-p)/m^2^, where *n* = required sample size, t = confidence level at 95% (standard value of 1.96), *p* = estimated prevalence of ED, m = margin of error at 5% (standard value of 0.05). We took estimated prevalence of ED as 25% based on a previous study [24]. Therefore, the calculated samples size was 288 and considering 10% of non-response rate the final sample size taken was 316.

### Data collection

Ethical approval for this study was obtained from the corresponding author’s university. Data were collected by three trained research assistants (RA). After written informed consent was obtained, the eligible participants were individually invited to fill in questionnaires independently. If they had difficulties completing the questionnaire, the RA would read each item aloud, give explanations without any inducement, and record their responses. The survey completion time ranged from 15 to 20 min. A total of 370 questionnaires were sent out, 352 participants’ data were finally included in the analysis, with 18 (4.9%) excluded due to incomplete data (*n* = 12) or over-centralized responses (*n* = 6).

## Measures

### Demographic variables

A self-reported personal information form was designed to collect demographic information. It included age, gender, spouse (yes/no), education level, religion, co-residents, major caregiver, health insurance, income source, and monthly income. The medical variables included type of tumor, surgery (yes/no), chemotherapy (yes/no), radiotherapy (yes/no), use of analgesic (yes/no), and course of disease (month). They were collected from the patients’ medical records.

### Independent variables

SPB was measured by the Chinese version of the Self-perceived Burden Scale (C-SPBS) [25]. It consists of eight items that are scored on a five-point Likert scale (1 as *none of the time*, 5 as *all of the time*). The total score of the scale ranges from 8 to 40, with a higher total score indicating higher SPB. Its Cronbach’s α is 0.874.

### Mediating variables

ED and anxiety were the two mediating variables in this study. The Existential Distress Scale (EDS) was used to measure ED of advanced cancer patients. It was first compiled by Lo et al. [26], and translated and validated in Chinese by our research team [27]. The Chinese EDS is a 10-item questionnaire covering three dimensions (meaninglessness, loneliness, and low self-worth). Each item is scored from 0 to 4 (0 as *unbearable distress*, 4 as *no distress*). A higher score represents a higher level of ED. The Cronbach’s α for the C-EDS is 0.892 and ranges from 0.747 to 0.858 for its dimensions.

The Hospital Anxiety and Depression Scale (HADS) was used to measure anxiety [28]. The 14-item self-reported scale was originally developed to indicate the possible presence of anxiety and depression. It contains two seven-item subscales: one for anxiety (HADS-A) and one for depression (HADS-D). The HADS-A was employed in this study to measure anxiety. Each item is scored on a four-point Likert scale (e.g., 0 = *as much as I always do*; 3 = *not at all*). The total HADS-A score ranges from 0 to 21, with a higher score indicating a higher probability of being screened for anxiety. The Cronbach’s α of the HADS-A ranges from 0.80 to 0.93.

### Dependent variable

QoL was assessed by the Quality-of-Life Concerns in the End of Life Questionnaire (QoLC-E), which contains 29 items [29]. One of the items was used to measure overall subjective QoL on a numeric scale ranging from 0 to 10 (0 as the worst, 10 as the best). The other 28 items cover eight subscales, namely physical discomfort, food-related concerns, health care concerns, support, negative emotions, sense of alienation, existential distress, and value of life. All items are rated on a four-point Likert scale (1 as *the least desirable*, 4 as *the most desirable*), with higher scores indicating a higher level of satisfaction with QoL. The Cronbach’s α for the scale is 0.87 and ranges from 0.57 to 0.83 for its subscales.

### Statistical analysis

Descriptive statistics were calculated for all outcome measures. Categorical variables were reported by frequency and percentage, and continuous variables by mean and standard deviation. Pearson correlation analyses were conducted for self-perceived burden, existential distress, anxiety, and QoL. Multiple and hierarchical regression analysis was used to confirm the influenced factors of QoL, which were controlled as covariates in mediating analysis.

Hayes’s bootstrapping method was used to examine the multiple mediation effects of SPB, ED, anxiety, and QoL [30]. Traditionally, Baron and Kenny’s causal steps approach was commonly used in mediation analysis; however, it has been criticized for its limitations, such as low statistical power. Compared with the causal steps approach, the production of coefficients approach (Sobel test) has more statistical power, but it requires the assumption of normality for the indirect effect sampling distribution, and the sampling distribution of indirect effect tends to be asymmetric, with non-zero skewness and kurtosis. The bootstrapping method is a non-parametric method for indirect effects, and its confidence intervals (CI) can better account for the irregularity of the sampling distribution of the indirect effect. As a result, bootstrapping has a more accurate CI, and yields a higher power than the causal steps approach and Sobel test. In our study, we generated 5000 bootstrap samples to produce bootstrap CI for the indirect effect. An indirect effect is assumed to be significant at an alpha level of 0.05 if its 95% CI does not include zero.

Model fit is determined by several goodness of fit indexes: the values of the Chi-Square (χ2) test, degrees of freedom (df), the comparative fit index (CFI), the Tucker–Lewis Index (TLI), and the root mean square error of approximation (RMSEA). The following values for an acceptable fit of the model were used: χ2/df < 5.00, TLI > 0.90, and CFI > 0.90 and RMSEA < 0.08 [31]. Descriptive analyses were conducted by IBM SPSS Statistics Version 25.1, and the path models were performed using Mplus Version 7.0.

## Results

### Demographic variables

Table 1 shows the demographic characteristics of 352 participants. More than half of the participants were male (64.8%), aged 18–59 years (54.8%), unaffiliated with a religion (51.7%), mainly cared for by a spouse (59.1%), and engaged in Resident health insurance (67.0%). The vast majority (93.2%) of the participants had a spouse, 47.1% of the participants’ educational level was primary school or below, and 38% of the participants’ monthly income was between $157 and $470. In terms of medical characteristics, over half of the participants were diagnosed as having a digestive tumor (60.5%), diagnosed as having cancer within the past 12 months (54.3%), and did not experience surgery treatment (51.1%). Most of the participants received chemotherapy (84.1%) and did not undergo radiotherapy (82.4%) or use analgesic (81.0%).

**Table 1:** Demographic characteristics of participants (*n* = 352)

Variable	Frequency (%)	Variable	Frequency (%)
**Gender**		**Age(years)**	
Male	228(64.8%)	18–59	193 (54.8%)
Female	124(35.2%)	≥ 60	159 (45.2%)
**Spouse**		**Religion**	
yes	328 (93.2%)	yes	170(48.3%)
no	24 (6.8%)	no	182(51.7%)
**Educational level**		**Major caregiver**	
Primary school or below	158(44.9%)	spouse	208(59.1%)
Middle school	89(25.3)	Adult child	76(21.6%)
High school and above	108(29.8%)	others	68(19.3%)
**Health insurance**		**Monthly income per person (US$)**	
Employee health insurance	110 (31.3%)	< 157	62(17.6%)
Resident health insurance	236(67.0%)	157–	134(38.0%)
others	6(1.7%)	471–	128(36.4%)
		942	28(8.0)
**Type of tumor**		**Course of disease (month)**	
digestive tumor	213(60.5%)	1–12	191(54.3%)
respiratory tumor	93(26.5%)	13–24	63(17.8%)
reproductive tumor	29(8.2%)	25–36	40(11.4%)
others	17(4.8%)	≥ 37	58(16.5)
**Surgery**		**Chemotherapy**	
yes	172(48.9%)	yes	296(84.1%)
no	180(51.1%)	no	56(15.9%)
**Radiotherapy**		**Use of analgesic**	
yes	62(17.6%)	yes	67(19.0%)
no	290(82.4%)	no	285(81.0%)

### Descriptive statistics and correlations among the main variables

Table 2 shows that the total scores of SPB, ED, anxiety, and QoL were 19.66 ± 7.75, 8.48 ± 7.12, 5.85 ± 4.21, and 81.69 ± 10.28 (Mean ± SD), respectively. All four variables were significantly correlated with one another. SPB was positively related to ED (*r* = 0.338, *p* < 0.01) and anxiety (r = 0.453, *p* < 0.01), while it was negatively related to QoL (*r* = -0.409, *p* < 0.01). ED was positively related to anxiety (*r* = 0.352, *p* < 0.01) and negatively related to QoL (*r* = 0–0.382, *p* < 0.01). Anxiety was negatively related to QoL (r = -0.663, *p* < 0.01).

**Table 2:** Descriptive statistics and correlations among the variables (*n* = 352)

Variables	M	SD	Correlations among variables			
			1	2	3	4
1. SPB	19.66	7.75	1.00	0.338^**^	0.453^**^	-0.409^**^
2. ED	8.48	7.12	-	1.00	0.352^**^	-0.382^**^
3. anxiety	5.85	4.21	-	-	1.00	-0.663^**^
4. QoL	81.69	10.28	-	-	-	1.00

^**^P < 0.01

### Mediating effects of existential distress and anxiety

**Fig. 2:**
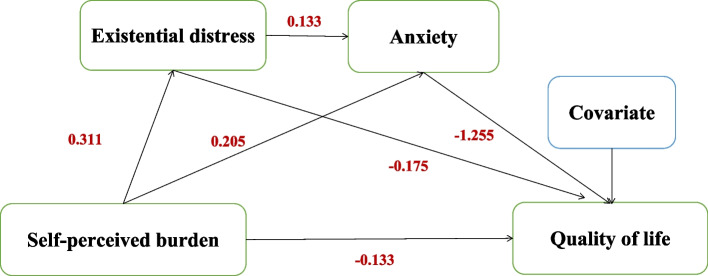
Results of final path model (*N* = 352). Note. Covariates for QoL were surgery, use of analgesic, and monthly income per person according to the result of multiple and hierarchical regression analysis of QoL (see Additional file 1)

The findings regarding the mediating effects of ED and anxiety on the relationship between SPB and QoL are shown in Table 3, and the final path model is presented in Fig. 2. According to the result, the final path model had acceptable fitting indices, X^2^ = 31.652, df = 6, *P* < 0.05, X^2^/df = 5.28; CFI = 0.938; TLI = 0.846; RMSEA < 0.08. The total effect of SPB on QoL was significant (b = -0.385, SE = 0.048, *p* < 0.001, percentile CI = [-0.472, -0.285]). The direct effect accounted for 26.75% of the total effect (b = -0.103, SE = 0.046, *p* = 0.026, percentile CI = [-0.194, -0.011]), and the mediating effect accounted for 73.25% of the total effect (b = -0.282, SE = 0.033, *p* < 0.001, percentile CI = [-0.353, -0.219]). Within the mediating effect, both ED and anxiety significantly mediated the relationship between SPB and QOL (b = -0.042, SE = 0.016, *p* = 0.008, percentile CI = [-0.075, -0.012]; b = -0.199, SE = 0.030, *p* < 0.001, percentile CI = [-0.266, -0.148]), and accounted for 14.89% and 70.57% of the mediating effect, respectively. The chain mediation of ED to anxiety was also significant and accounted for 14.54% of the total mediation effect (b = -0.041, SE = 0.012, *p* = 0.001, percentile CI = [-0.068, -0.020]).

**Table 3:** Mediating effects of ED and anxiety on the relationships between SPB and QoL (*n* = 352)

	**Point estimate**	**Product of coefficient**		**Bootstrapping**	
		**SE**	**P**	**Percentile CI**	
				**Lower**	Upper
**Total Effects**					
SPB——QoL	-0.385	0.048	< 0.001	-0.472	-0.285
**Direct effects**					
SPB——QoL	-0.103	0.046	0.026	-0.194	-0.011
**Indirect effects**					
**Total Indirect Effects**	-0.282	0.033	< 0.001	-0.353	-0.219
SPB——ED——QoL	-0.042	0.016	0.008	-0.075	-0.012
SPB——Anxiety——QoL	-0.199	0.030	< 0.001	-0.266	-0.148
SPB——ED——Anxiety——QoL	-0.041	0.012	0.001	-0.068	-0.020

## Discussion

The present study confirms Chen’s conceptual model of ED and Walster’s equity of theory in cancer patients, indicating that ED and anxiety partially mediate the relationship between SPB and QoL among patients with advanced cancer [7, 8]. Moreover, ED has direct effects on anxiety, and sequentially on QoL. This study provides new evidence for explaining how SPB affects the QoL of advanced cancer patients.

Our study has found that SPB was negatively associated with QoL, and the direct effects of SPB on QoL account for 26.75% of the variance in QoL among advanced cancer patients. This fact has been supported by various studies [10, 12, 32, 33]. Patients with advanced cancer may be dependent on caregivers in their daily life. However, it is difficult for them to restore the balance between given and received due to their deteriorating illness [8]. According to the equity theory [8], the inability to reciprocate affects their psychological well-being in the form of frustration, worry, guilt, or SPB. It also causes patients to less willing to ask for help. In this way, patients are reducing the benefit and inputs from caregivers, such as physical care, symptom management, and emotional support [10, 34, 35]. Consequently, QoL is further negatively affected.

Existential issues have increased in importance since cancer patients became seriously ill. Our mediating model showed that the relationship between SPB and QoL can be mediated by ED, confirming the concept framework of ED proposed by Chen et al. [7].They have revealed that SPB is one important antecedent of ED in cancer patients, and ED can further cause poor QoL. Dependence on caregivers makes patients with advanced cancer usually feel themselves a physical, economic, and emotional burden on their family. Such perceived sense of being a burden on others can result in patients’ ED, which involves meaninglessness, loss of autonomy, loss of dignity, hopelessness, and death anxiety [7]. ED has been identified to be associated with worse physical, functional, and psychological well-being among cancer patients by previous study [24], which may account for its impact on QoL. Ghiggia’s [15] study further indicated that ED is a significant predictor of a diminished QoL of cancer patients.

The present study has also revealed that anxiety played a mediating role between SPB and QoL in patients with advanced cancer. In other words, SPB may arise their anxiety and subsequently decrease QoL. The negative impact of SPB on anxiety can be explained by the equity theory [8], which holds that loss balance in giving and receiving may result in negative psychological reaction. It can be also possible explained by patients’ views on interpersonal relationships. In Asia culture, the interpersonal relationships function is under the assumption of mutual obligation and responsibility [36]. The behaviors of advanced cancer patients being no longer able to fulfill mutual obligations and responsibilities contradicts their views in relationship, provoking anxiety accordingly [36]. Cumulative evidence showed that anxiety contributes to reducing QoL in advanced cancer patients [16, 37]. It involves in worsening physical symptoms, impairment in social and cognitive functioning and perceived impairment in global health status [16]. Moreover, anxiety has been demonstrated as a risk factor of poor QoL [17].

Our study also has first disclosed that 14.54% of the total indirect effects were explained by the serial mediation effects of ED and anxiety on QoL, indicating that the SPB could affect QoL via the path of ED to anxiety. ED is characterized as a person’s incapacity to cope effectively with a stressful event [13]. According to Parle et al., a failure coping with stress would lead to negative mental adjustment [38]. Anxiety is one of demonstration of negative mental adjustment during the process of disease [38]. In our study, advanced cancer patients ED felt subjectively incompetence and uncertain, they could not cope with the threaten from their disease, and then anxiety arose. Thus, ED can exert anxiety and serially impair QoL. It implies additional attention should be placed on alleviating ED and anxiety for improving QoL among advanced cancer patients.

## Limitations

This study has some limitations. First, the cross-sectional design does not allow us to draw causal inferences. Second, a convenience sampling procedure may limit the generalization of the findings, as nearly half of the current sample was poorly educated. Third, the sole use of self-reported measures may result in social desirability bias, influencing the interpretation of this study’s findings. Future studies can adopt a longitudinal or experimental design with mixed methods involving interviews to further verify the present study findings.

### Relevance to clinical practice

Given the life-threatening nature of cancer, how to conduct end-of-life care and improve patients' QoL is always an essential concern for health providers [39, 40]. This study provides new insights into the impact of SPB on QoL. It also suggested that to improve QoL, health providers should place more emphasis on releasing ED and anxiety of patients. Specifically, health providers should consider a multifaceted approach to relieve patients' ED, such as developing strategies to restore their autonomy, meaning of life, hope, dignity, and so on. It is also crucial for health providers to minimize anxiety; targeted programs can focus on enhancing patients' coping abilities with cancer, such as helping them promote realistic goals achievement.

## Conclusions

To our best knowledge, this is the first study to explore the mediating effects of ED and anxiety between the relationship between SPB and QoL in China. The results suggest that SPB can directly negatively predict QoL, and it can also predict QoL indirectly by three paths: (1) the mediating role of ED, (2) the mediating role of anxiety, (3) the chain mediating role of ED and anxiety. Therefore, health providers should consider a multifaceted approach to reduce ED and anxiety for improving QoL.

## Supplementary Information

**Additional file 1.** 

## Data Availability

The datasets used or analysed during the current study are available from the corresponding author on reasonable request.

## Abbreviations

SPB: Self-perceived Burden
ED: Existential Distress
QoL: Quality of life
KPS: Karnofsky Performance Status
RA: Research assistants
C-SPBS: The Chinese version of the Self-perceived Burden Scale
C-EDS: The Chinese version of the Existential Distress Scale
HADS-A: The Hospital Anxiety and Depression Scale for Anxiety
QOLC-E: Quality-of-Life Concerns in the End of Life Questionnaire
CI: Confidence Intervals
χ2: The values of the Chi-Square
df: Degrees of freedom
CFI: The comparative fit index
TLI: The Tucker–Lewis Index
RMSEA: The root mean square error of approximation
M: Mean
SD: Standard deviation; SE:Standard error
