# Supplementary material for: Self-perceived burden predicts lower quality of life in advanced cancer patients: the mediating role of existential distress and anxiety
Source: BMC Geriatr. 2022 Oct 17;22:803. doi: 10.1186/s12877-022-03494-6 (PMC9575302; doi:10.1186/s12877-022-03494-6)
Supplement: Supplementary file 1 — Additional file 1. [file 12877_2022_3494_MOESM1_ESM.doc]

Appendix1

Table 1 Result of multiple and hierarchical regression analysis of QoL (n=352)

| predictors | b | SE b | β | t | P |
| --- | --- | --- | --- | --- | --- |
| surgery | -2.703 | 1.023 | -0.132 | -2.643 | 0.009 |
| use of analgesic | -8.715 | 1.362 | -0.333 | -6.399 | 0.000 |
| monthly income per person | 4.757 | 2.298 | 0.125 | 2.070 | 0.039 |
